# Metabolic proteins with crucial roles in *Edwardsiella tarda* antioxidative adaptation and intracellular proliferation

**DOI:** 10.1128/msystems.00391-23

**Published:** 2023-09-20

**Authors:** Xinhui Wang, Boguang Sun

**Affiliations:** 1 CAS and Shandong Province Key Laboratory of Experimental Marine Biology, Institute of Oceanology, Chinese Academy of Sciences, Qingdao, China; 2 CAS Center for Ocean Mega-Science, Chinese Academy of Sciences, Qingdao, China; 3 Laboratory for Marine Biology and Biotechnology, National Laboratory for Marine Science and Technology (Qingdao), Qingdao, China; California State University, Stanislaus, Turlock, California, USA

**Keywords:** *Edwardsiella tarda*, oxidative stress, virulence, metabolic reprogramming, proteomics

## Abstract

*Edwardsiella tarda* is a severe fish pathogen, featured by its capacity to live inside host phagocytes. For intracellular survival, it is crucial for *E. tarda* to neutralize the deleterious effect of host reactive oxygen species (ROS). Accumulating evidence suggests that bacterial metabolism is closely connected to oxidative resistance. However, the roles of *E. tarda* metabolic proteins in antioxidative adaptation and intracellular proliferation remain elusive. In this study, we performed a proteomic analysis on *E. tarda* and identified 111 proteins responsive to H_2_O_2_-mediated oxidative stress. Based on this data, we further obtained eight crucial proteins, including seven metabolic proteins, for *E. tarda* antioxidation and intracellular infection. Among them, two C4-dicarboxylate transporters were found necessary for *E. tarda* to disseminate in fish tissues. Furthermore, the substrate of the two transporters was identified as L-aspartate, which was proven to be essential for the full antioxidative capacity of *E. tarda*. Our results indicate that reprogramming the metabolic flux to the production of pyruvate, a ketoacid capable of neutralizing ROS, was likely a pivotal strategy of *E. tarda* to survive the oxidative environments inside host cells. Together, the findings of this study highlight the significance of metabolic reprogramming for bacterial redox homeostasis and intracellular infection.

**Importance:**

*Edwardsiella tarda* is a significant fish pathogen that can live in challenging environments of reactive oxygen species (ROS), such as inside the phagocytes. Metabolic reconfiguration has been increasingly associated with bacterial oxidative tolerance and virulence. However, the metabolic proteins of *E. tarda* involved in such processes remain elusive. By proteomic analysis and functional characterization of protein null mutants, the present study identified eight crucial proteins for bacterial oxidative resistance and intracellular infection. Seven of them are metabolic proteins dictating the metabolic flux toward the generation of pyruvate, a key metabolite capable of scavenging ROS molecules. Furthermore, L-aspartate uptake, which can fuel the pyruvate generation, was found essential for the full antioxidative capacity of *E. tarda*. These findings identified seven metabolic proteins involved in bacterial oxidative adaptation and indicate that metabolic reprogramming toward pyruvate was likely a pivotal strategy of bacteria for antioxidative adaptation and intracellular survival.

## INTRODUCTION

*Edwardsiella tarda* is a Gram-negative facultatively anaerobic bacterium, belonging to the *Enterobacteriaceae* family (1, 2). It naturally inhabits freshwater, estuary, and marine environments and can infect various animal hosts, ranging from fish, reptiles, birds to mammals (3, 4). *E. tarda* causes a severe fish disease termed edwardsiellosis and leads to significant economic losses in aquaculture worldwide (3). *E. tarda* is intractable to conventional antibiotic treatment, largely due to its capacity for intracellular survival and proliferation within host cells including phagocytes (4, 5).

Professional phagocytes, such as macrophages, neutrophils, and dendritic cells, are featured by their capacities for microbial phagocytosis, an exquisitely regulated process enabling the engulfment and destruction of the invading pathogens within a plasma membrane-derived vacuole termed phagosome (6, 7). Phagocytes possess a vast and sophisticated arsenal of antimicrobial weapons, including a cascade-activated protein machinery allowing phagosome maturation and acidification, antimicrobial proteins and peptides such as cathepsins and cation antimicrobial peptides, and reactive nitrogen and oxygen species (7).

Reactive oxygen species (ROS) production, termed respiratory burst, is a common programmed response of phagocytes upon bacterial infection (8). NADPH oxidase is the major driving force of ROS generation in phagosome (9). It is a protein complex composed of the integral membrane subunits, i.e., gp91^phox^ and p22^phox^, forming flavocytochrome b558, and the cytosolic subunits, i.e., p40^phox^, p47^phox^, and p67^phox^ (10). Upon immune stimulation, the cytosolic subunits bind to flavocytochrome b558 and assemble together with small GTPases Rac1 and Rac2 to transfer electrons from NADPH to molecular oxygen and generate O_2_^·−^ (7). Within the phagosomal lumen, O_2_^·−^ is dismutated to form the highly reactive hydrogen peroxide (H_2_O_2_), which can be converted into hydroxyl radicals by the Fe^2+^-dependent Fenton reaction with superoxide dismutase (SOD) or participate in the generation of hypochlorous acid and chloramines (7, 11). These highly toxic ROS molecules enable the effective killing of intraphagosomal microorganisms through various mechanisms, such as disrupting metabolism by damaging iron-sulfur enzymes, creating DNA damage and accumulated mutagenesis, and inactivating proteins through carbonylation (12 – 14).

Intracellular pathogenic bacteria have evolved an array of strategies to counteract host ROS (7). A common mechanism shared by diverse bacteria for ROS evasion is by the production of SOD, which catalyzes the conversion of the O_2_^·−^ to H_2_O_2_. The latter can be further detoxified by conversion to O_2_ and H_2_O with bacterial catalases (10). In *E. tarda*, deletion of the coding gene for an iron-cofactored superoxide dismutase resulted in significantly decreased bacterial resistance to macrophage-mediated killing, in parallel with the significantly enhanced respiratory burst of fish macrophages (15). The genome of *E. tarda* harbors at least two catalase-encoding genes, i.e., *katB* and *katG* (16). KatB is essential for bacterial resistance against killing by host phagocytes, while KatG confers resistance against exogenous H_2_O_2_ (17, 18). In addition, DNA binding protein from starved cells (Dps), thioredoxin H (TrxH), ferric uptake regulator (Fur), hemolysin activator (Eha), and type III secretion system effector, EseJ, are known factors implicated in ROS evasion strategy of *E. tarda* (19 – 23). Apart from the specialized antioxidant enzymes and their mediators for ROS elimination, mounting evidence has pointed out that bacterial metabolism is intricately linked to antioxidative defense (24 – 26). For instance, ketoacids, such as α-ketoglutarate, pyruvate, and glyoxylate, are able to consume oxidizing agents via non-enzymatic decarboxylation and generate organic acids; therefore, the enzymes and transporters that dictate the metabolic currency toward the synthesis of these metabolites are vital for the bacteria to maintain the cellular redox homeostasis (25). However, in *E. tarda*, the engagement of metabolites, metabolic enzymes and transporters, and metabolic network in bacterial adaptation to oxidative stress and survival in phagocytes remains to be delineated.

In this work, we performed a comparative proteomic analysis to systematically screen for the proteins involved in the antioxidative defense of *E. tarda* and identified eight proteins including seven metabolic proteins as crucial participants in the antioxidative adaptation and intracellular proliferation. Our results suggest that metabolic reprogramming for the production of antioxidant metabolites is likely a pivotal survival strategy for *E. tarda* to survive and thrive in oxidative environments.

## RESULTS

### Identification of differentially abundant proteins induced by oxidative stress

**Fig 1:**
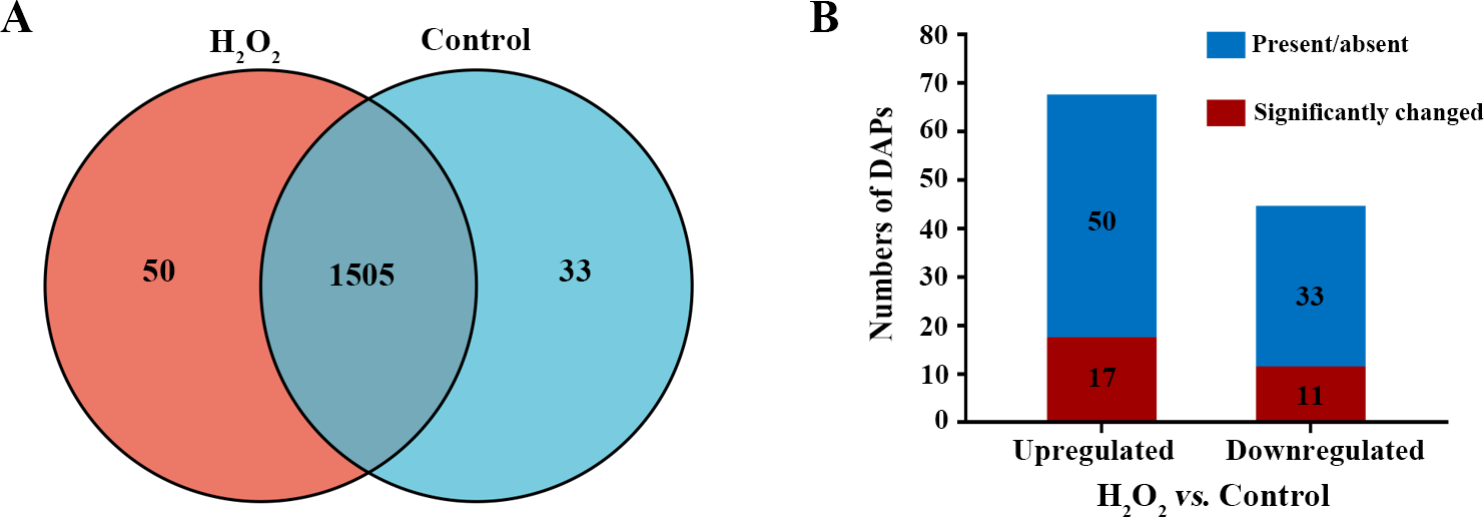
Analysis of the proteins identified and quantified by proteomics analysis. (**A**) Intersection analysis of the proteins of the H_2_O_2_ group and the control group. (**B**) Summary of the differentially abundant proteins in the comparison of H_2_O_2_ group vs control group.

In order to identify the proteins that are involved in the oxidative defense of *E. tarda*, comparative proteomics analysis was performed with 10 mM of H_2_O_2_ as the redox stressor, which had a potent impact on bacterial growth (Fig. S1). A total of 1,588 proteins were identified in the H_2_O_2_ treatment group (1,555 proteins) and the control group (1,538 proteins), of which 1,505 were shared in common (Fig. 1A).

Using a screening criteria of fold change ≥2 and *P*-value < 0.05, 111 differentially abundant proteins (DAPs) were identified in the comparison of H_2_O**_2_** group vs control group, 67 of which were upregulated and 44 were downregulated (Fig. 1B; Table S3 and S4). Fifty of the upregulated proteins were detected only in the H_2_O_2_ group (Fig. 1B; Table S4), while 33 of the downregulated proteins were found only in the control group (Fig. 1B; Table S4).

### GO and KEGG analyses of the DAPs

**Fig 2:**
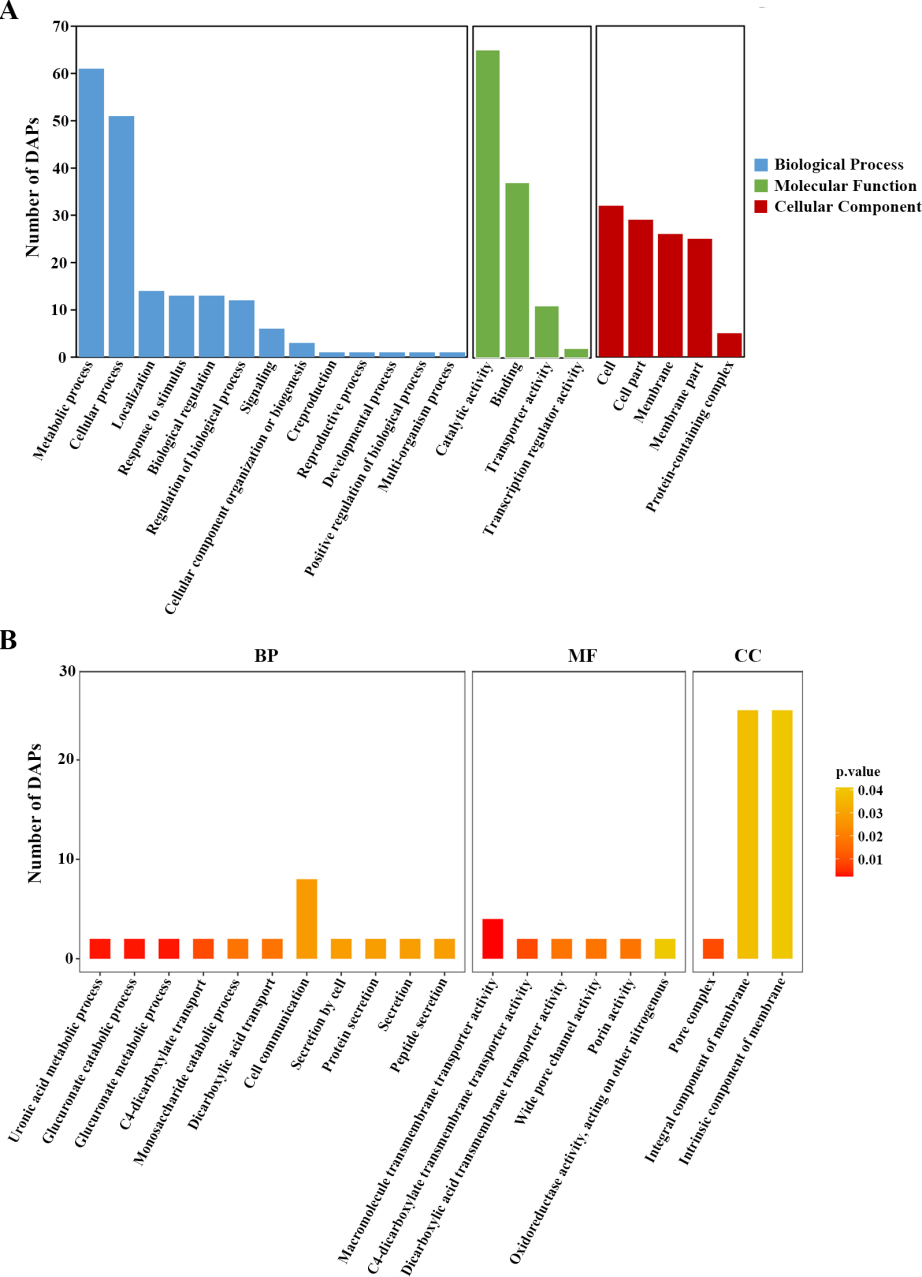
GO classification and enrichment analysis of DAPs in the H_2_O_2_ group vs the control group. (**A**) Functional classification of DAPs by GO analysis. (**B**) GO enrichment (top 20) of the DAPs in the H_2_O_2_ group vs control group. The *P*-value was set as <0.05.

Gene ontology (GO) analysis showed that the DAPs of H_2_O_2_ vs control were classified into three categories: biological process (BP), molecular function (MF), and cellular component (CC) (Fig. 2A). The significantly enriched BP terms were glucuronate metabolic process, uronic acid metabolic process, glucuronate catabolic process, C4-dicarboxylate transport, monosaccharide catabolic process, dicarboxylic acid transport, cell communication, secretion by cell, secretion, protein secretion, and peptide secretion (Fig. 2B; Table S5). The significantly enriched MF terms were macromolecule transmembrane transporter activity, C4-dicarboxylate transmembrane transporter activity, wide pore channel activity, dicarboxylic acid transmembrane transporter activity, porin activity, and oxidoreductase activity, acting on other nitrogenous compounds as donors (Fig. 2B; Table S5). The significantly enriched CC terms were pore complex, integral component of membrane, and intrinsic component of membrane (Fig. 2B; Table S5).

Kyoto Encyclopedia of Gene and Genome (KEGG) analysis was conducted to reveal the signaling pathways contributing to the oxidative defense of *E. tarda* (Fig. S2). The DAPs were categorized into the top 10 KEGG pathways of pentose and glucuronate interconversions, arginine biosynthesis, pantothenate and CoA biosynthesis, tryptophan metabolism, valine, leucine and isoleucine biosynthesis, biosynthesis of vancomycin group antibiotics, one carbon pool by folate, polyketide sugar unit biosynthesis, acarbose and validamycin biosynthesis, and two-component system (Table S6).

### Identification of DAPs transcriptionally responsive to oxidative stress

To identify the DAPs that are transcriptionally responsive to oxidative stress, the top 10 upregulated DAPs (ranked based on the proteomic fold change) and the top 25 DAPs that were detected only in the H_2_O_2_ group (ranked based on the proteomic abundance) were analyzed for their relative mRNA levels in the H_2_O_2_ group vs the control group (Table S3 and S4). The results showed that the expressions of ETAE_0310 (anaerobic C4-dicarboxylate transporter [CDT], DcuA), ETAE_0790 (C4-dicarboxylate transporter, DcuC1), ETAE_1753 (dihydromonapterin reductase), ETAE_1821 (starvation-inducible DNA-binding protein Dps), ETAE_2289 (mannonate dehydratase), ETAE_2291 (fructuronate reductase), ETAE_2977 (putative dicarboxylate-binding periplasmic protein), and ETAE_3323 (glycerate 2 kinase) were significantly upregulated in the H_2_O_2_ group, compared to the control group (Table 1). These eight genes were the potential key proteins involved in the oxidative defense (KPODs) of *E. tarda* and, therefore, subjected to further investigation.

**TABLE 1:** List of the DAPs transcriptionally responsive to oxidative stress

Protein ID	Gene ID	Annotation	Proteomic fold change (H_2_O_2_ vs control)	Transcriptional fold change (H_2_O_2_ vs control)
ACY83157.1	ETAE_0310	Anaerobic C4-dicarboxylate transporter, DcuA	+∞^*a*^	2.583 ± 0.059^*b*^
ACY83635.1	ETAE_0790	C4-dicarboxylate transporter, DcuC1	+∞^*a*^	2.708 ± 0.015^*b*^
ACY84590.1	ETAE_1753	Dihydromonapterin reductase/dihydrofolate reductase	+7.39^*c*^	2.791 ± 0.122^*b*^
ACY84658.1	ETAE_1821	Starvation-inducible DNA-binding protein, Dps	+9.82^*b*^	3.035 ± 0.216^*b*^
ACY85124.1	ETAE_2289	Mannonate dehydratase	+2.82^*b*^	2.901 ± 0.221^*b*^
ACY85126.1	ETAE_2291	Fructuronate reductase	+13.51^*c*^	4.686 ± 0.144^*b*^
ACY85810.1	ETAE_2977	Putative dicarboxylate-binding periplasmic protein	+2.30^*b*^	3.131 ± 0.178^*b*^
ACY86154.1	ETAE_3323	Glycerate 2 kinase	+∞^*a*^	2.027 ± 0.074^*b*^

^*a*^ Proteomic DAPs detected only in the H_2_O_2_ group.

^*b*^ *P* < 0.01.

^*c*^ *P* < 0.05.

### The roles of KPODs in the antioxidation and intracellular proliferation of *E. tarda*

**Fig 3:**
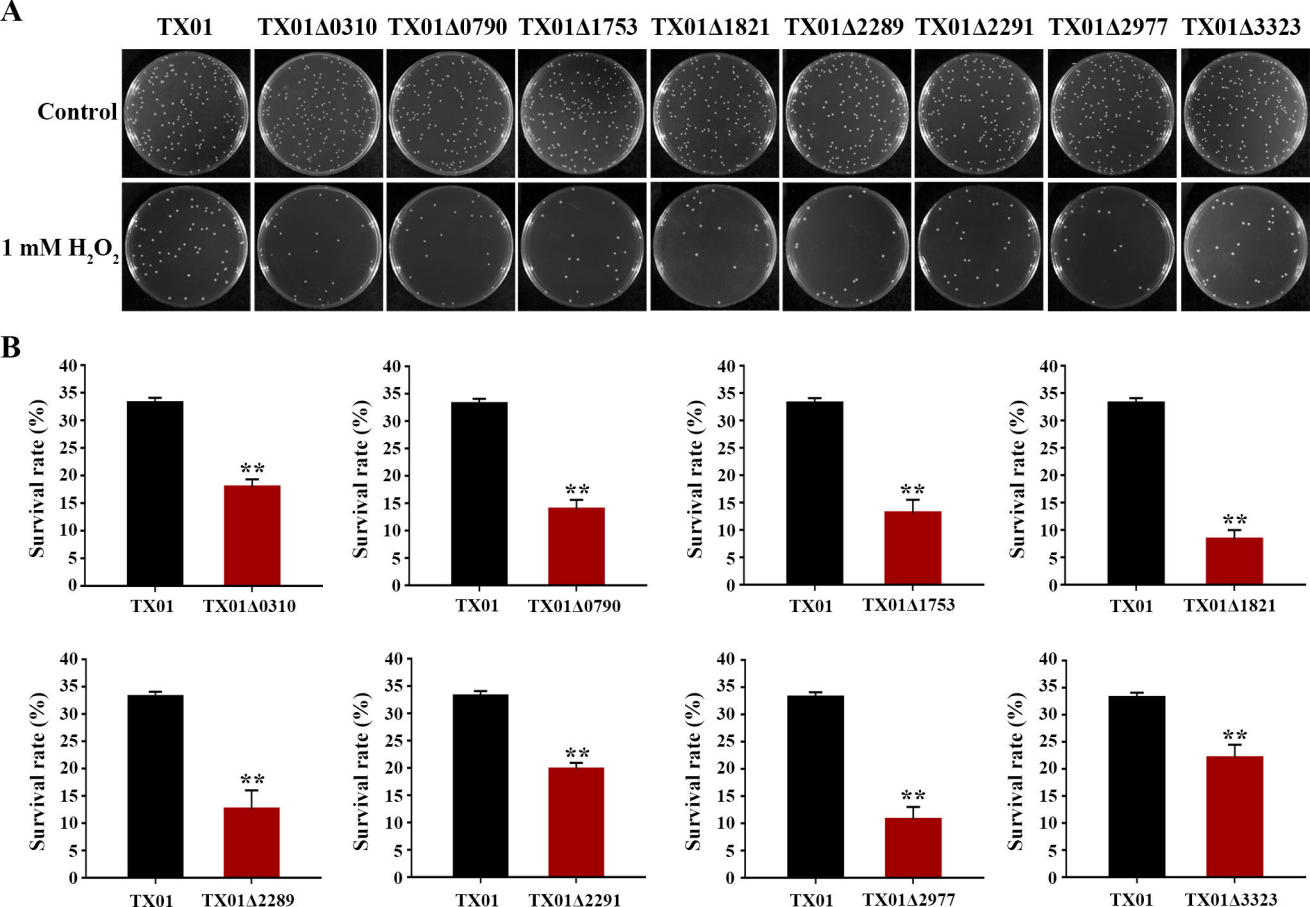
The effect of gene deletion of each KPOD protein on the survival of *E. tarda* under H_2_O_2_ stress. TX01, TX01Δ0310, TX01Δ0790, TX01Δ1753, TX01Δ1821, TX01Δ2289, TX01Δ2291, TX01Δ2977, and TX01Δ3323 were incubated with 1 mM H_2_O_2_ for 2 h, then the bacterial cells were plated on LB plates and incubated at 28°C for 24 h. The number of CFU was counted (**A**) and bacterial survival rate (**B**) was determined. Data are the means of triplicates and are shown as means ± SD, *n* = 3. **P < 0.01.

**Fig 4:**
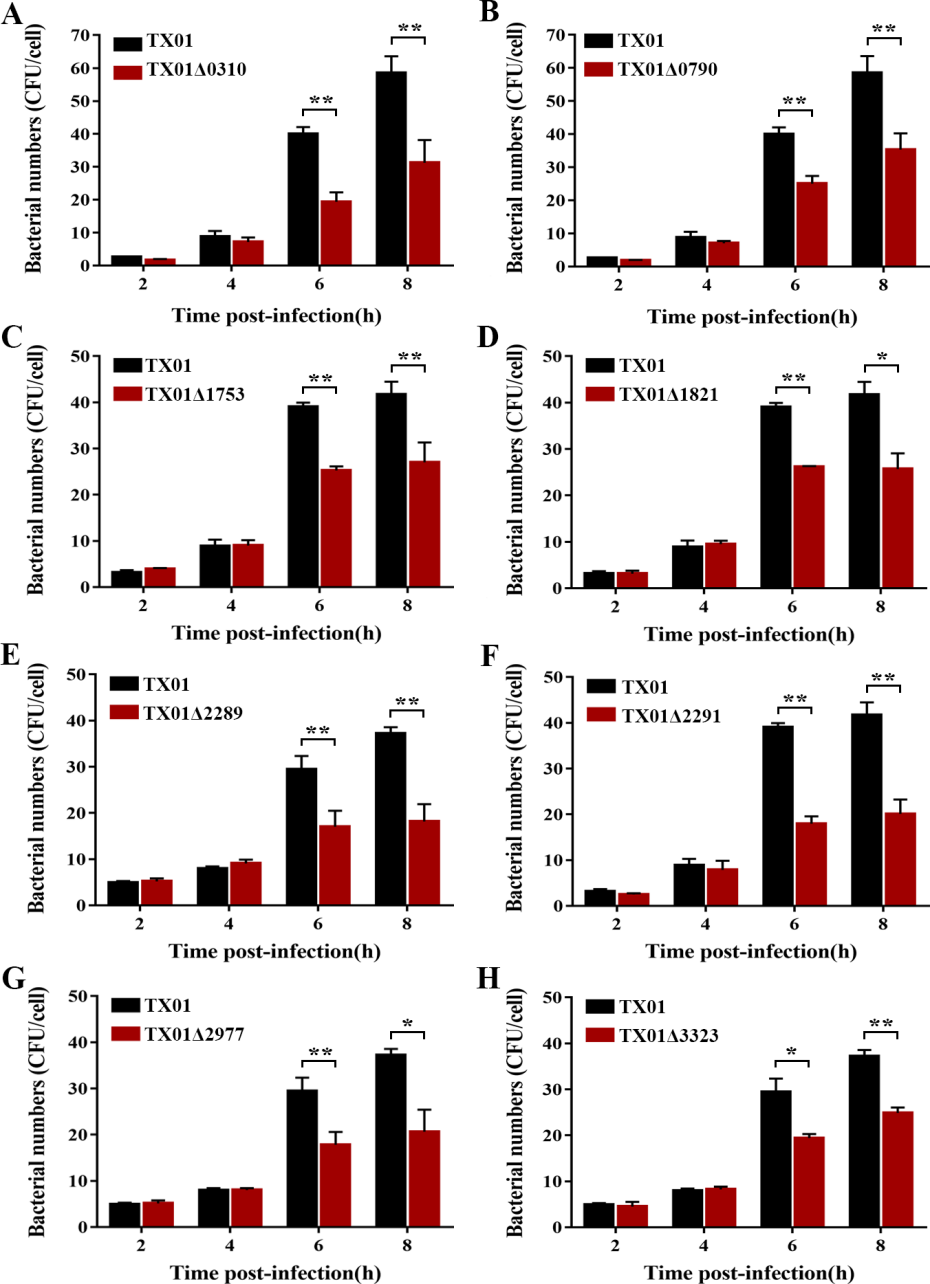
The effect of gene deletion of each KPOD protein on the capacity of *E. tarda* for intracellular proliferation. RAW264.7 cells were infected with TX01, TX01Δ0310 (**A**), TX01Δ0790 (**B**), TX01Δ1753 (**C**), TX01Δ1821 (**D**), TX01Δ2289 (**E**), TX01Δ2291 (**F**), TX01Δ2977 (**G**), and TX01Δ3323 (**H**) for 1.5 h, and the extracellular bacteria were killed by antibiotic treatment. The cells were incubated for indicated time periods. Then the number of intracellular bacteria was determined by plate count. Data are the means of triplicates and are shown as means ± SD, *n* = 3. *P < 0.05, **P < 0.01.

In order to understand the role of KPOD proteins in the antioxidant capacity of *E. tarda,* the isogenic mutants of wild-type strain TX01, namely TX01Δ0310, TX01Δ0790, TX01Δ1753, TX01Δ1821, TX01Δ2289, TX01Δ2291, TX01Δ2977, and TX01Δ3323, were created by markerless gene deletion. The essentiality of KPOD proteins for the resistance of *E. tarda* to oxidative stress was assessed by H_2_O_2_ pulsing assay. The result showed that, under H_2_O_2_ stress, the survival rates of the mutants were all significantly reduced, in comparison with that of the wild-type TX01 (Fig. 3). The requirement of KPOD proteins for intracellular proliferation of *E. tarda* was evaluated by cellular-infection assay using macrophages, within which the bacteria must neutralize the deleterious effects of ROS to survive and thrive. The results indicated that, at 4 hpi and 6 hpi, the intracellular bacterial numbers of all the mutant strains were remarkedly less than the wild-type TX01 (Fig. 4). These results suggest that all the eight KPOD proteins exerted crucial roles in the antioxidative defense and intracellular proliferation of *E. tarda*.

### The essentiality of KPOD CDTs for *E. tarda* to proliferate in the host

**Fig 5:**
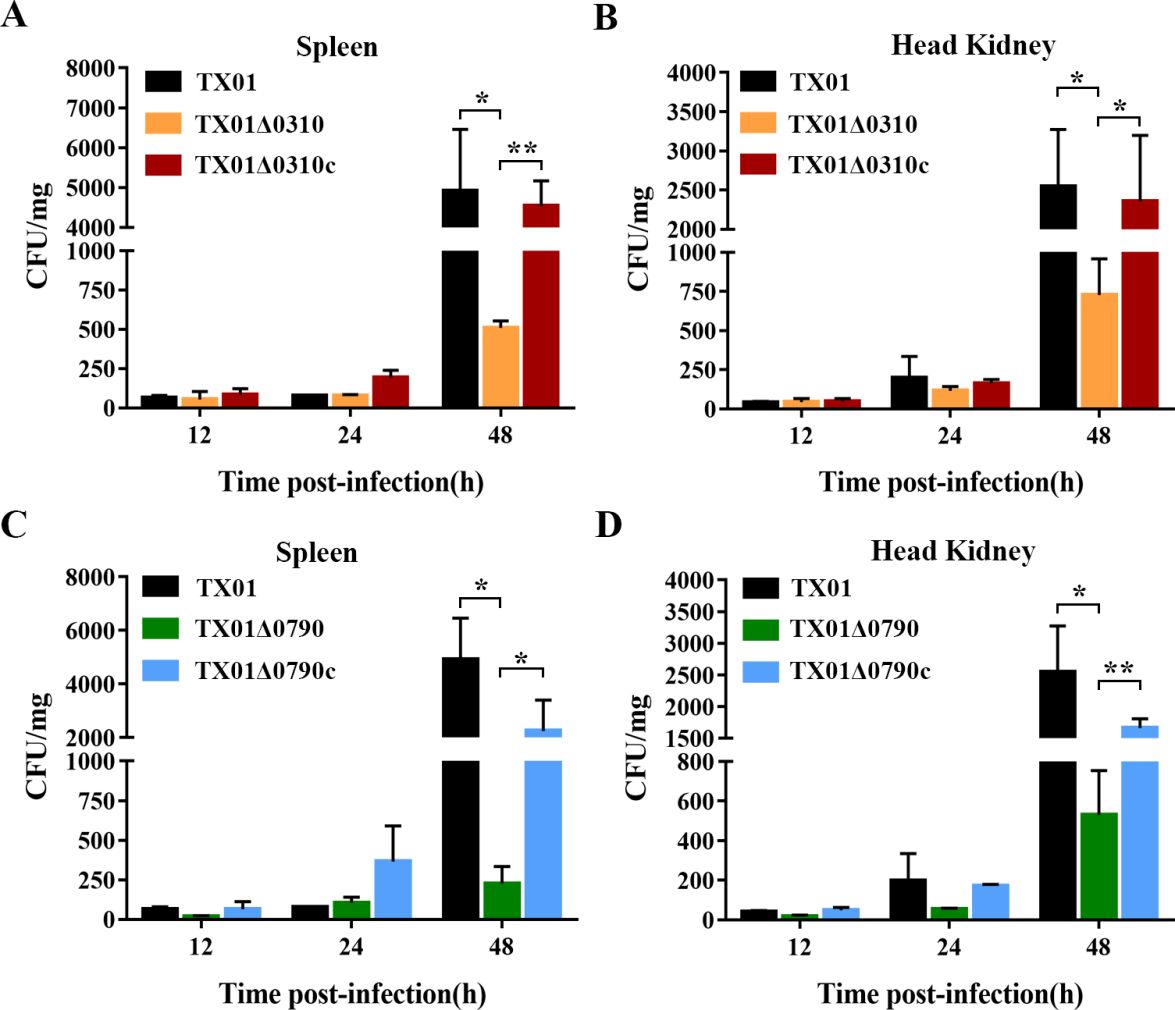
The effects of gene deletion of KPOD CDTs on *in vivo* proliferation of *E. tarda*. Turbot were infected with TX01, TX01Δ0310, and TX01Δ0310c (**A and B**), TX01Δ0790 and TX01Δ0790c (**C and D**). At 12, 24, and 48-h post-infection, bacterial numbers in the spleen (**A and C**), and head kidney (**B and D**) were determined by plate count. Data are the means of triplicates and are shown as means ± SD, *n* = 3. *P < 0.05, **P < 0.01.

Three of the eight KPOD proteins, namely anaerobic C4-dicarboxylate transporter, DcuA (ETAE_0310), C4-dicarboxylate transporter, DcuC1 (ETAE_0790), and putative dicarboxylate-binding periplasmic protein (ETAE_2977), are involved in C4-dicarboxylate transportation. To investigate the potential role of CDTs in the infectivity of *E. tarda*, we evaluated the capacities of TX01Δ0310 and its complementary strain TX01Δ0310c, TX01Δ0790 and its complementary strain TX01Δ0790c, as well as the wild-type strain TX01 for proliferation in the tissues of turbot, a natural fish host of *E. tarda*. Our results revealed that, compared to the control groups, both TX01Δ0310- and TX01Δ0790-infected groups had significantly lower bacterial loads in spleen and head kidney, respectively, at 48-h post-infection (Fig. 5), indicating that CDTs are essential for *E. tarda* to proliferate in host tissues.

### The substrate of CDTs and its role in the antioxidation of *E. tarda*

**Fig 6:**
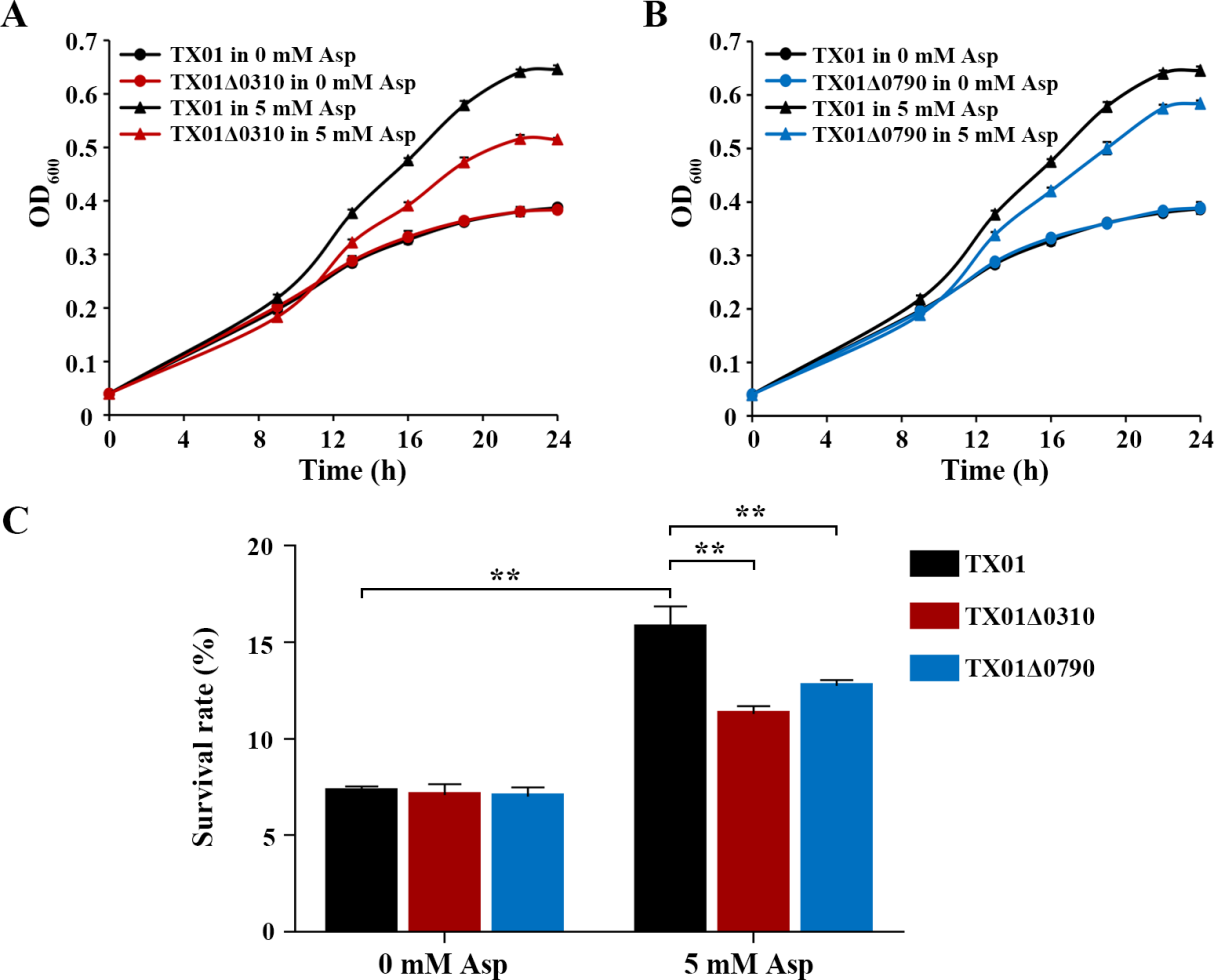
Identification of the substrate of KPOD CDTs and its role in antioxidation of *E. tarda*. Growth curves of *E. tarda* TX01, TX01Δ0310 (**A**), and TX01Δ0790 (**B**) were determined in FMCM medium without or with L-aspartic acid (Asp). *E. tarda* TX01, TX01Δ0310, and TX01Δ0790 were grown in FMCM medium without or with Asp and treated with 1 mM H_2_O_2_ for 2 h; then, the survival rates were determined by plate count (**C**). Data are the means of triplicates and are shown as means ± SD, *n* = 3. **P < 0.01.

In order to determine the possible substrate(s) transported by CDTs, growth curves of TX01Δ0310 and TX01Δ0790 were, respectively, compared with those of the wild-type TX01 in a chemically defined medium, with or without the potential substrates of Dcu family, namely L-aspartate, malate, or fumarate. The results indicated that in the absence of L-aspartate both TX01Δ0310 and TX01Δ0790 showed similar growth curves with TX01, while in the presence of L-aspartate both TX01Δ0310 and TX01Δ0790 exhibited notably delayed growth compared to that of TX01 (Fig. 6A and B). In contrast, in the presence of malate or fumarate, the growth curve of TX01Δ0310 or TX01Δ0790 was similar to that of TX01 (Fig. S3). These results suggest that the gene products of ETAE_0310 and ETAE_0790 are likely two transporters for L-aspartate.

To investigate the possible role of L-aspartate in the antioxidative capacity of *E. tarda*, we conducted H_2_O_2_ pulsing assay to evaluate the resistance of TX01, TX01Δ0310, and TX01Δ0790 to oxidative stress in the medium without or with L-aspartate. The results showed that in the absence of L-aspartate, the survival rates of TX01, TX01Δ0310, and TX01Δ0790 were similar (Fig. 6C). Whereas the addition of 5 mM L-aspartate remarkedly enhanced the survival rate of TX01, which was also significantly higher than that of TX01Δ0310 or TX01Δ0790 in the presence of L-aspartate (Fig. 6C). These results suggest that the uptake of L-aspartate was essential for the antioxidative capacity of *E. tarda*.

## DISCUSSION

*E. tarda* resides inside the host phagocyte, a challenging environment rife with deleterious ROS, and, therefore, requires efficient detoxification strategies for survival and thriving (3, 5, 23). In this study, we profiled the proteomic response of *E. tarda* to the oxidative stress induced by H_2_O_2_, a central molecule in the conversion chain of ROS, and identified eight proteins responsive to H_2_O_2_ stress both in translation and in transcription. Deletion of the coding genes for the eight proteins, respectively, resulted in significantly reduced capacity against H_2_O_2_ stress *in vitro* and remarkedly decreased proliferation in host macrophages, suggesting they are pivotal participants in antioxidative adaptation and intracellular proliferation of *E. tarda*. These proteins include seven metabolic enzymes and transporters, i.e., fructuronate reductase (ETAE_2291), mannonate dehydratase (ETAE_2289), glycerate 2 kinase (ETAE_3323), C4-dicarboxylate transporter DcuA (ETAE_0310), C4-dicarboxylate transporter DcuC1 (ETAE_0790), putative dicarboxylate-binding periplasmic protein (ETAE_2977), and dihydromonapterin reductase (ETAE_1753), and one non-metabolic protein, i.e., starvation-inducible DNA-binding protein, Dps (ETAE_1821).

In *Escherichia coli*, fructuronate reductase and mannonate dehydratase are important for glucuronate assimilation (27). Once inside the bacterial cell, glucuronate is first isomerized to D-fructuronate. Fructuronate reductase catalyzes the reduction of D-fructuronate to D-mannonate. Then, mannonate dehydratase catalyzes the dehydration of D-mannonate to 2-keto-3-deoxy-D-gluconate, which can be further phosphorylated to 2-keto-3-deoxy-D-gluconate-6-phosphate and fluxed to the Entner-Doudoroff pathway for the production of pyruvate (27, 28). In addition, glycerate 2 kinase catalyzes the phosphorylation of glycerate to 2-phosphoglycerate, which can also be fluxed to pyruvate production through the glycolysis pathway (29). In the present work, we found that fructuronate reductase, mannonate dehydratase, and glycerate 2 kinase were responsive to H_2_O_2_ stress and were part of the antioxidant mechanism of *E. tarda*. It is intriguing that all three of these metabolic enzymes can be linked to the generation of pyruvate, a ketoacid by chemical nature. It has emerged that ketoacids, including pyruvate, α-ketoglutarate, and glyoxylate, are able to function as ROS scavengers via non-enzymatic decarboxylation (25). Notably, the product of mannonate dehydratase KPG is also a ketoacid, which may be an unrevealed molecule with a significant role in the antioxidative defense of *E. tarda* and other bacteria.

In this work, we identified three C4-dicarboxylate transporting associated proteins, i.e., C4-dicarboxylate transporter DcuA, C4-dicarboxylate transporter DcuC1, and a putative dicarboxylate-binding periplasmic protein, as part of the antioxidant arsenal of *E. tarda*. In *Enterobacteriaceae*, C4-dicarboxylates such as fumarate, succinate, and malate, as well as C4-dicarboxylic amino acid L-aspartate can be uptake by C4-dicarboxylate transporters and utilized as nutrients and energy source (30, 31). Functional examination of DcuA and DcuC1 suggests that they are likely transporters for L-aspartate in *E. tarda* and are essential for this bacterium to proliferate inside macrophage and disseminate in host tissues. In addition, we found that L-aspartate is required for the full capacity of *E. tarda* for oxidative resistance. Bacteria assimilate aspartate to fuel the tricarboxylic acid (TCA) cycle through oxaloacetate or fumarate (32, 33). It was noted that the expression or activity of bacterial enzymes that metabolize TCA intermediates (such as oxaloacetate and malate) to pyruvate was heightened during oxidative metabolism (32, 34). In accordance with that, *Pseudomonas fluorescens* undergoes a metabolic reconfiguration to enhance pyruvate production upon H_2_O_2_ challenge (35). Notably, extraction of the intermediates from TCA cycle has bonus benefits for bacteria to alleviate the oxidative burden, as interrupted TCA would reduce the generation of NADH, the driving force of the electron transfer chain. The latter is a major site of intrinsic ROS production in bacterial cells (25). The above observations and our findings implicate that reprogramming the metabolic flow to the production of pyruvate may be a common strategy utilized by different bacteria to combat oxidative stress.

In this study, we identified dihydromonapterin reductase as a key contributor to the capacities of *E. tarda* for oxidative detoxification and intracellular survival. Dihydromonapterin reductase catalyzes the reduction of dihydromonapterin to tetrahydromonapterin (36). In bacteria, tetrahydromonapterin serves as an essential cofactor of the nitric oxide (NO) synthases (37). In *Bacillus subtilis*, NO produced by the bacterial NO synthase confers critical resistance to ROS attack by blocking the enzymatic release of free cysteines that fuel the generation of hydroxyl radicals by Fenton reaction and by directly reactivating catalase (38). In *E. tarda*, the roles of tetrahydromonapterin, bacterial NO synthase, and NO in the antioxidative defense remain to be delineated.

The only one non-enzymatic antioxidative protein identified in the present work is starvation-inducible DNA-binding protein, Dps, which is a ferritin-like protein being able to sequester iron to prevent the damaging Fenton reaction (39). In *E. tarda,* two Dps proteins, i.e., Dps1 and Dps2, have been characterized and found to be essential for mitigating the respiratory burst of macrophage and required for bacterial dissemination *in vivo* (19). The present work discovered a third Dps (ETAE_1821), which was upregulated upon H_2_O_2_ stress and involved in the antioxidative mechanism of *E. tarda*.

In conclusion, in this study, we profiled the proteomic alteration of *E. tarda* in response to the oxidative stress mediated by H_2_O_2_ treatment and identified eight key proteins for bacterial redox homeostasis, seven of which are metabolic enzymes and transporters. We then revealed the importance of L-aspartate uptake in the oxidative resistance of *E. tarda*. These findings indicated that metabolic reprogramming is likely a pivotal strategy of *E. tarda* for tolerance of oxidative stress and survival in the hostile environments of the host.

## MATERIALS AND METHODS

### Bacterial strains

The bacterial strains used in this study are listed in Table S1. *Edwardsiella tarda* TX01 and derivative strains were grown in Luria-Bertani (LB) medium at 28°C (40). For protein function assay, a chemically defined medium was formulated based on the FMC medium by Terleckyj et al. (41), with modifications as follows: supplementation of 110 µg/mL D-methionine and omitting glucose and L-aspartate from the recipe. The medium was named FMCM. Where indicated, 5 mM L-aspartate, malate, or fumarate was added to the medium. *Escherichia coli* DH5α and S17-1 λ*pir* were grown in LB medium at 37°C. All bacterial cultures were initiated from a single colony on LB agar. Where required, polymyxin B, ampicillin, and chloramphenicol were supplemented at concentrations of 50, 100, and 30 µg/mL, respectively.

### Cell culture

RAW264.7 cells (American Type Culture Collection, Rockville, MD, USA) were cultured at 37°C in Dulbecco’s modified Eagle’s medium (Corning, Arizona, USA) supplemented with 10% (vol/vol) fetal bovine serum (Gibco, Grand Island, NY, USA), 100 units/mL penicillin, and 100 µg/mL streptomycin (Solarbio, Beijing, China) in a humidified atmosphere containing 5% CO_2_.

### Fish

Healthy turbot (*Scophthalmus maximus*), averaging 20 g, were purchased from Hai-Shuo-Jia-Yuan aquaculture company in Qingdao city, Shandong province, China. The fish were maintained at ~20°C in aerated seawater and fed with commercial feed daily. Turbot were acclimatized in the laboratory conditions for 2 weeks before the experiments.

### Preparation of the proteomic samples

*E. tarda* TX01 was grown in LB broth at 28°C until the OD_600_ ≈ 0.5. Then, the culture was added with 0 mM (as control) or 10 mM hydrogen peroxide (H_2_O_2_). After 3 h, the bacteria were centrifuged and washed with phosphate buffered saline (PBS) at 4°C, and frozen in liquid nitrogen for subsequent experiments.

A total of six samples (three samples in the control group and three samples in the H_2_O_2_ treatment group) were lysed with SDT (4% SDS, 100 mM Tris-HCl, 1 mM DTT, pH 7.6) buffer. The protein of each sample was extracted and quantified with the BCA Protein Assay Kit (Bio-Rad, USA). Subsequently, trypsin digestion of protein was performed according to the method of filter-aided sample preparation procedure described by Matthias Mann (42). Then, the digested samples were desalted, concentrated, and reconstituted in 40 µL of 0.1% (vol/vol) formic acid.

### LC-MS/MS analysis

Label-free proteomic analysis was performed by the Shanghai Applied Protein Technology Co. Ltd. (Shanghai, China) (43). In brief, liquid chromatography coupled to tandem mass spectrometry (LC-MS/MS) was performed on a Q Exactive mass spectrometer (Thermo Scientific) that was coupled to an Easy nLC HPLC liquid system (Thermo Scientific). Digested samples were loaded onto a reverse phase trap column (Acclaim PepMap100, 100 µm × 2 cm, nanoViper C18, Thermo Scientific) connected to the C18-reversed phase analytical column (Easy Column, 10 cm long, 75 µm inner diameter, 3 µm resin, Thermo Scientific) in buffer A (0.1% formic acid) and separated with a linear gradient of buffer B (84% acetonitrile and 0.1% formic acid) at a flow rate of 300 nL/min. The mass spectrometer was operated in positive-ion mode. The parameters of primary MS were set as follows: MS data survey scan range, 300–1,800 m/z; resolution, 70,000 at 200 m/z; automatic gain control target, 1e6; maximum inject time, 50 ms; and dynamic exclusion duration, 60.0 s. The parameters of secondary MS were set as follows: resolution for HCD spectra, 17,500 at m/z 200; isolation width, 2 m/z; normalized collision energy, 30 eV; and underfill ratio, 0.1%.

### Proteins identification, quantitation and bioinformatics analysis

The MS raw data were combined and searched against the reverse NCBI_*Edwardsiella tarda* EIB202_6916_20201010 database using the MaxQuant software (1.5.3.17), as previously described (44). The search criteria were as follows: full tryptic specificity was required, two missed cleavage was allowed, carbamidomethyl (C) was set as the fixed modifications, the oxidation (M) was set as the variable modification, the MS/MS tolerances were set at 20 ppm, the protein and peptide false discovery rate was set to 0.01, and peptides only assigned to a given protein group were considered as unique.

The statistical significance was determined with an unpaired Student’s *t*-test, *P* < 0.05, and |fold change| ≥ 2 was set as the screening criteria to identify the DAPs. The Venn diagram analysis of the proteins was performed using the platform of Shanghai Applied Protein Technology Co., Ltd (http://cloud.aptbiotech.com/#/main-page). The GO annotation and the KEGG pathway of DAPs were annotated using the software program Blast2GO and KEGG Automatic Annotation Server. After Fisher’s exact test, GO terms and pathways with *P*-value < 0.05 were considered to be significantly enriched.

### qRT-PCR assay

Aliquots of bacterial samples prepared for the proteomic analysis were lysed using Bacteria RNA Extraction Kit (Vazyme, Nanjing, China). RNA was extracted using Bacterial RNA Kit (Omega Bio-Tek, Guangzhou, China), with an optional on-membrane DNase I treatment step included to remove the residual genomic DNA. cDNA was obtained by reverse transcription using the RevertAid First Strand cDNA Synthesis Kit (Thermo Fisher Scientific, MA, USA). Quantitative real-time reverse transcription PCR (qRT-PCR) was performed in technical duplicates (three biological replicates for a treatment group) using the ChamQ Universal SYBR qPCR Master Mix (Vazyme, Nanjing, China) in a QuantStudio 3 Real-Time PCR System. Relative transcription was quantified by the comparative Ct (2^-ΔΔCT^) method with *topA* as an internal control (45). All primers are listed in Table S2.

### Construction of the gene-deleted mutant and complementary strains of *E. tarda*

In-frame gene deletion was carried out as reported previously (46). In brief, the upstream and downstream DNA fragments of each target gene were amplified and overlapped by PCR and then inserted into pDM4 (47) at the *Bgl*II site by homologous recombination using ClonExpressII One Step Cloning Kit (Vazyme, Nanjing, China). The resulting plasmids were introduced into *E. coli* S17-1 λ*pir* and then *E. tarda* TX01 by conjugative transfer. The mutant strains were generated by two-step homologous recombination and verified by PCR and sequencing (Table S1).

For the construction of the ETAE_0310 or ETAE_0790 complementary strain, the entire coding sequence of ETAE_0310 or ETAE_0790 was amplified by PCR and then inserted into pBT3 (48) at the *Eco*RV site by homologous recombination. The resulting plasmid was introduced into the mutant strain TX01Δ0310 or TX01Δ0790 by electroporation, yielding the complementary strain TX01Δ0310c and TX01Δ0790c.

All primers used in this study are listed in Table S2.

### H_2_O_2_ pulsing assay

*E. tarda* strains were grown in LB medium at 28°C until OD_600_ ≈ 0.5. The bacteria were collected by centrifugation, washed with PBS, and suspended in PBS with 0 or 1 mM H_2_O_2_ to a final concentration of 1 × 10^5^ CFU/mL. After 2-h incubation at 28°C, the cells were diluted in PBS and plated on LB agar plates. The plates were incubated at 28°C for 24 h; the number of colony-forming units was counted and bacterial survival was determined as follows: (number of survived cells after H_2_O_2_ treatment/number of recovered cells without H_2_O_2_ treatment) × 100%.

To examine the effects of exogenous metabolite on the resistance of *E. tarda* to H_2_O_2_ pulsing, TX01, TX01Δ0310, and TX01Δ0790 were grown in FMCM medium at 28°C until OD_600_ ≈ 0.2, and then 5 mM or 0 mM (as control) of L-aspartate was added to the medium. The bacteria were grown for another 2 h at 28°C, then subjected to H_2_O_2_ pulsing and plate count as described above.

### Cellular infection assay

RAW264.7 cells were infected with *E. tarda* wild-type TX01 and derivates as described previously with slight modification (49). Briefly, *E. tarda* strains were prepared as above and resuspended in Opti-MEM (Gibco, Grand Island, NY, USA) to a final concentration of 1 × 10^7^ CFU/mL. Bacteria were added to RAW264.7 cells in a 24-well plate at a multiplicity of infection of 10:1. The plates were centrifuged at 800 *g* for 10 min and incubated at 30°C for 1.5 h. Then, the culture medium was replaced by fresh Opti-MEM containing 400 µg/mL gentamicin. The plates were incubated at 30°C for 40 min for the antibiotics to kill the extracellular bacteria. The cells were then washed three times with PBS and cultured in Opti-MEM containing 20 µg/mL gentamicin for 0, 2, 4, and 6 h to allow intracellular proliferation of the bacteria. At each time point, 600 µL 1% (vol/vol) Triton X-100 was added to the plate to lyse the cells; the lysate was diluted and plated onto LB agar. The plates were incubated at 28°C for 24 h, and the number of colonies was counted.

### *In vivo* infection assay

*In vivo* infection was performed as described previously with slight modification (46). Briefly, TX01, TX01Δ0310, TX01Δ0310c, TX01Δ0790, and TX01Δ0790c were prepared as above and resuspended in PBS to a concentration of 1 × 10^7^ CFU/mL. Turbot were randomly divided into five groups (nine fish/group) and infected via intramuscular injection with 100 µL TX01, TX01Δ0310, TX01Δ0310c, TX01Δ0790, or TX01Δ0790c. At 12-, 24-, and 48-h post-infection, fish were euthanized with an overdose of tricaine methanesulfonate (Sigma-Aldrich, Madrid, Spain). The spleen and kidney were collected aseptically, homogenized, and serially diluted in PBS. The diluted homogenates were plated onto LB plates. The plates were incubated at 28°C for 24 h, and the number of colonies was counted.

### Statistical analysis

All experiments were performed three times. Statistical analyses were carried out with GraphPad Prism version 7.00 (GraphPad Software Inc., San Diego, CA, USA). Data were analyzed with Student’s *t*-test. Statistical significance was defined as *P* < 0.05.

## Data Availability

All data of this article have been provided in the main body or supplemental material. The proteomic data are provided in Table S3 and Table S4.

## SUPPLEMENTAL MATERIAL

Supplemental material msystems.00391-23-s0001.docx Figures S1 to S3; Tables S1, S2, S5, and S6; legends for Tables S3 and S4.

Table S3 msystems.00391-23-s0002.xlsx List of the significantly changed DAPs.

Table S4 msystems.00391-23-s0003.xlsx List of the DAPs detected only in H_2_O_2_ group or control group.

ASM does not own the copyrights to Supplemental Material that may be linked to, or accessed through, an article. The authors have granted ASM a non-exclusive, world-wide license to publish the Supplemental Material files. Please contact the corresponding author directly for reuse.
