# Supplementary material for: Metabolic proteins with crucial roles in Edwardsiella tarda antioxidative adaptation and intracellular proliferation
Source: mSystems. 2023 Sep 20;8(5):e00391-23. doi: 10.1128/msystems.00391-23 (PMC10654080; doi:10.1128/msystems.00391-23)
Supplement: Supplemental material — Figures S1 to S3; Tables S1, S2, S5, and S6; legends for Tables S3 and S4. [file msystems.00391-23-s0001.docx]

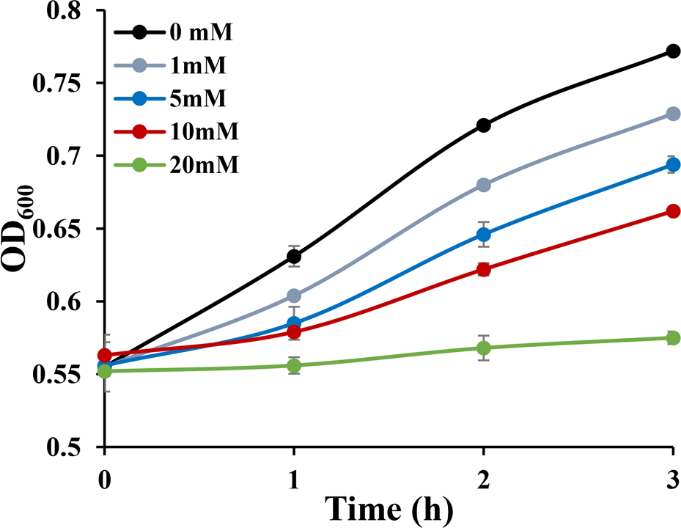


**FIG S1** Growth curves of *E. tarda* under the stress of H_2_O_2_. *E. tarda* was grown in LB medium at 28 °C until the OD_600_ ≈ 0.5, then different concentrations of H_2_O_2_ were added to the medium for indicated time periods. Data are the means of triplicates and are shown as means ± SD, n=3.


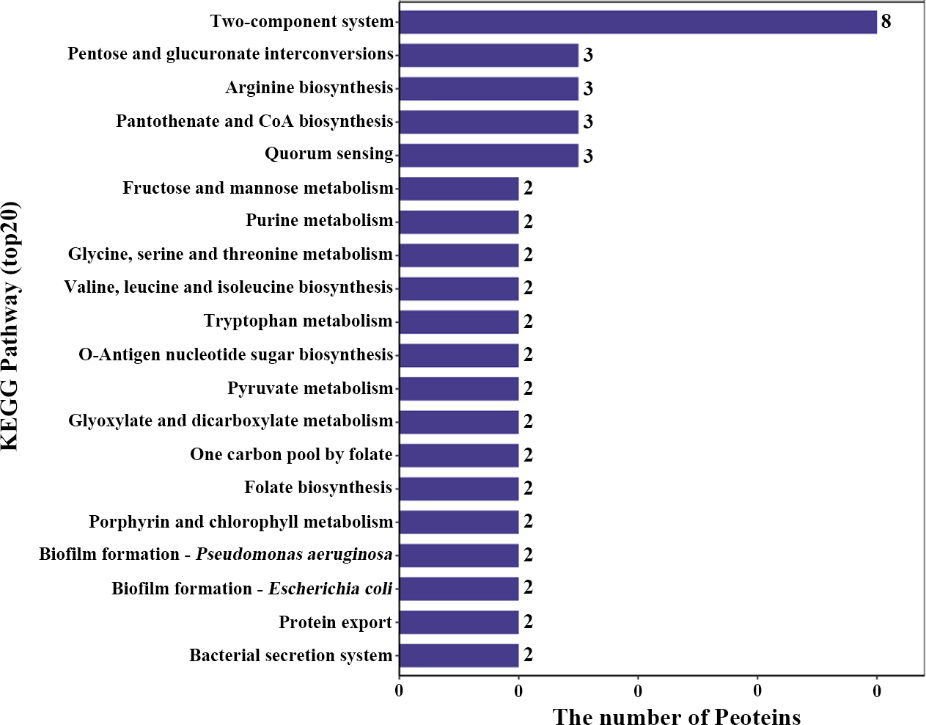


**FIG S2** Top 20 enriched KEGG pathways of the DAPs in H_2_O_2_ group *vs*. control group.


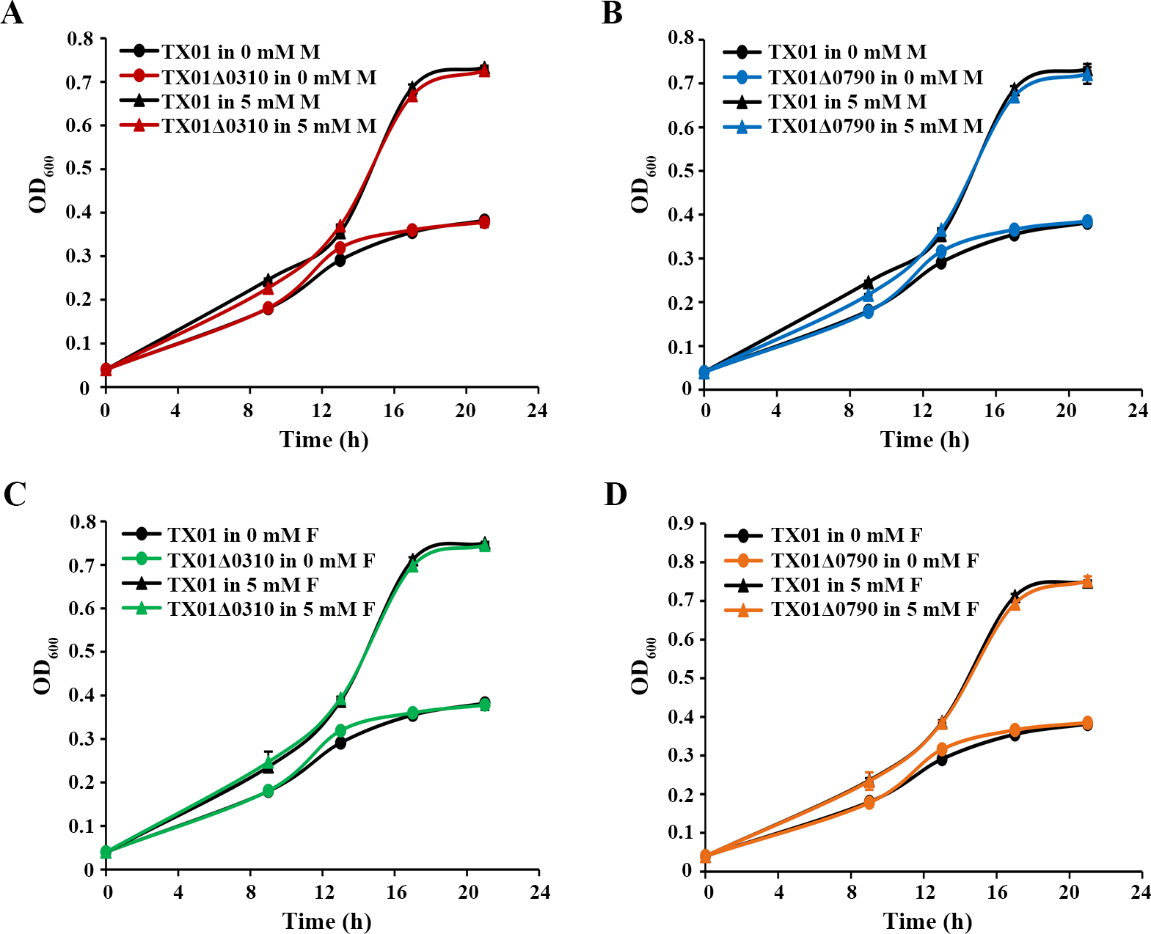


**FIG S3** Effect of malate or fumarate on the growth of *E. tarda*. Growth curves of *E. tarda* TX01, TX01Δ0310 (A and C), and TX01Δ0790 (B and D) were determined in FMCM medium without or with malate (A and B) or fumarate (C and D). Data are the means of triplicates and are shown as means ± SD, n=3. M, malate. F, fumarate.

**TABLE S1** Bacterial strains and plasmids used in this study.

| **Strain and plasmid** | **Description^1^** | **Reference or source** |
| --- | --- | --- |
| **Strain** |  |  |
| *Edwardsiella tarda* |  |  |
| TX01 | Wild type strain, PMB^r^ |  |
| TX01Δ0310 | TX01, with the ETAE_0310 gene deleted, PMB^r^ | This study |
| TX01Δ0790 | TX01, with the ETAE_0790 gene deleted, PMB^r^ | This study |
| TX01Δ1753 | TX01, with the ETAE_1753 gene deleted, PMB^r^ | This study |
| TX01Δ1821 | TX01, with the ETAE_1821 gene deleted, PMB^r^ | This study |
| TX01Δ2289 | TX01, with the ETAE_2289 gene deleted, PMB^r^ | This study |
| TX01Δ2291 | TX01, with the ETAE_2291 gene deleted, PMB^r^ | This study |
| TX01Δ2977 | TX01, with the ETAE_2977 gene deleted, PMB^r^ | This study |
| TX01Δ3323 | TX01, with the ETAE_3323 gene deleted, PMB^r^ | This study |
| TX01Δ0310c | TX01Δ0310 harboring pBT3-0310, PMB^r^, Amp^r^ | This study |
| TX01Δ0790c | TX01Δ0790 harboring pBT3-0790, PMB^r^, Amp^r^ | This study |
| *Escherichia coli* |  |  |
| DH5α | Cloning host | Tsingke, China |
| S17-1 λ*pir* | Cloning host for pDM4 and derivates | Biomedal, Seville, Spain |
| **Plasmid** |  |  |
| pDM4 | A suicide plasmid for gene knockout, Cm^r^ | (47) |
| pBT3 | An expression plasmid for the construction of complementary strains, Amp^r^ | (48) |
| pBT3-0310 | pBT3 containing the ETAE_0310 gene, Amp^r^ | This study |
| pBT3-0790 | pBT3 containing the ETAE_0790 gene, Amp^r^ | This study |

^1^ PMB, polymyxin B; Amp, ampicillin; Cm, chloramphenicol.

**TABLE S2** Primers used in this study.

| **Primer** | **Sequence (5’ - 3’)** | **Source** |
| --- | --- | --- |
| 2291RTF | GGCATCGCCTGTGAACCCTT | This study |
| 2291RTR | GGCTACCGTTGAGCATACGCA | This study |
| 1821RTF | CTCTGGACGCCACTCTGACGA | This study |
| 1821RTR | GTGCGCCTGCTTAGTCAGCA | This study |
| 1753RTF | GACGCTCAATGCCATGCTGCA | This study |
| 1753RTR | GCGGCATAGGCGATATGGCTA | This study |
| 2072RTF | CTGACGTTGGACAAGCGCAA | This study |
| 2072RTR | CCCAGTATGCTCAGGCCGT | This study |
| 2289RTF | CGCAGAGGATTACAGCACGGA | This study |
| 2289RTR | CAATACCGTCATACTCCGCCAG | This study |
| 1826RTF | GCTCAGCGCGCCAACAACCT | This study |
| 1826RTR | CGGAGCGCCATCCCAGCT | This study |
| 1975RTF | CAAGAGATTGACAGGGCTATCAAGGA | This study |
| 1975RTR | CCCTTTACGCTCAGCATCCGT | This study |
| 0436RTF | GGATTACTTTGCCGCCCACGGTT | This study |
| 0436RTR | CAGCACGGCCGTCTTGAACTGA | This study |
| 2861RTF | GTTCGGTAACCCTGAGACCACCA | This study |
| 2861RTR | GCCACCTTGTTCTTGACCACCT | This study |
| 2977RTF | CCGCCTACGTCGCCGGTA | This study |
| 2977RTR | CCGCCCATCAGCTCCACCAT | This study |
| 1755RTF | CCCTGATGCTGCTTATCGGACT | This study |
| 1755RTR | GAGGTGCTGTCGAAGTGCGT | This study |
| 2737RTF | GCTGCTGATCCAAATCCACGGT | This study |
| 2737RTR | CTGTGGTGCCATTGACGGTA | This study |
| 3323RTF | CCCTACATCGCTATGGCACGCT | This study |
| 3323RTR | CCGGGACGCAAACGAGCATTCA | This study |
| 0274RTF | GCTGCGCATCTGGATGCAGT | This study |
| 0274RTR | CAGGATGAACGCCAGCAGCA | This study |
| 0445RTF | CTGGCGCTGCTGGAGCAACA | This study |
| 0445RTR | CACGCCAGAGAGGTATGCGTCT | This study |
| 3409RTF | CTTGGCCATGTATCAGAGAAGCT | This study |
| 3409RTR | CGCCGCAGTCAATCCGCA | This study |
| 0459RTF | GACGATGTGGCGCACTGCCT | This study |
| 0459RTR | CTCACCCGCAGGCGAACCCA | This study |
| 2717RTF | CGATCGGCCAACAACCTGCTGA | This study |
| 2717RTR | GCAGGGTCGCCACCTCGTCA | This study |
| 1527RTF | GTCCACCGGCGCACGCCTCA | This study |
| 1527RTR | GGCCAGGATGATCCCCGGTAA | This study |
| 3112RTF | GATGCGCAGCGCCAGTTCGT | This study |
| 3112RTR | GCGCCAGATTGCTAAAGCCCA | This study |
| 1803RTF | GAGAGCGCACGTTTATCAGCGT | This study |
|  |  |  |
| **TABLE S2** Continued | |  |
| 1803RTR | CGGCAGGGCATACATCCATTC | This study |
| 1506RTF | CCGGTGCTGTGCTACGGCTTA | This study |
| 1506RTR | GACGCAGCACCATATTCGCCTT | This study |
| 3241RTF | GCCGGATCGCTTTTCAGGAGAT | This study |
| 3241RTR | GTGCGCCATCATGGAGGGCT | This study |
| 0505RTF | GGCGACGGAGCAGCTCTCTA | This study |
| 0505RTR | CAATCTACGCCGACCACCGA | This study |
| 0144RTF | CATTCAGCACACGACCTTCGGT | This study |
| 0144RTR | GGCGTCAGCAGCATCCAGA | This study |
| 1194RTF | GATGCCGATGGCGATCCTATTTCA | This study |
| 1194RTR | CCGACGGCCTTCATCAAAGCA | This study |
| 2845RTF | CGCAGGCTATGGTCGTCAGA | This study |
| 2845RTR | GTGTTCTTGGTGAGCTGATAGGT | This study |
| 0790RTF | GACGGTGCTGGTGGTGGGCATT | This study |
| 0790RTR | GGAGCCGACCGCCATCAGAATC | This study |
| 2904RTF | CATCGTCTGTCGCGGCACCCT | This study |
| 2904RTR | GCGCAGATTGTGGCTCTGGC | This study |
| 0650RTF | GGTGGTGCCATTGCTGATCGA | This study |
| 0650RTR | GGTCTGGGCGGCCAGGATA | This study |
| 1502RTF | GTATCCGCAGTGTCATCCCGA | This study |
| 1502RTR | CTCCGTCGCCGAAATCACCATT | This study |
| 0310RTF | CACCGATCTCTGCCGCCGTGGT | This study |
| 0310RTR | CCCTCTTCCAGACGCTTGCGGT | This study |
| 1492RTF | GGTGGTGGATGAGCCCTTTGA | This study |
| 1492RTR | GGATGGCAAACAGCCCGTGA | This study |
| 2597RTF | CCTACCTGACGCTGGGAGT | This study |
| 2597RTR | CGTCCAGGGAATAAAGGGACGA | This study |
| 0105RTF | CCTCCAAGGCCGCCAGCGAT | This study |
| 0105RTR | GCCAGCGCATTGATGATGGTCA | This study |
| TopARTF | GCTATGAGGTAGAAGAGG | (45) |
| TopARTR | CCCATATACTTGCCAAAG | (45) |
| 0310upF | GGTTACCCGCATGCAAGATCTCTCTGACTGCGGTTCCTACGTGAT (BglⅡ) | This study |
| 0310upR | TTAAAGACGCCCAGTCTGGCCCCCAAGA | This study |
| 0310downF | GACTGGGCGTCTTTAACCACCCGTTCTT | This study |
| 0310downR | GGGGCCCTTCTAGATAGATCTCTGATGGGGCGTAATCCGGATCT (BglⅡ) | This study |
| 0790upF | GGTTACCCGCATGCAAGATCTCCCACGGCCCGGTCAAGGT (BglⅡ) | This study |
| 0790upR | ACCACCTCATAGCCGCGCAGGATCAGGC | This study |
| 0790downF | GCGGCTATGAGGTGGTGCGGCGTACCTC | This study |
| 0790downR | GGGGCCCTTCTAGATAGATCTCGCCTCATCCGCACGCCCT (BglⅡ) | This study |
| **TABLE S2** Continued | |  |
| 1753upF | GGTTACCCGCATGCAAGATCTCCAGCGTTTATTACACGATGCGGA (BglⅡ) | This study |
| 1753upR | ATGAAACGGGTGATCACCACCGGCGGC | This study |
| 1753downF | TGATCACCCGTTTCATTACCGGTCGTACCA | This study |
| 1753downR | GGGGCCCTTCTAGATAGATCTGTTAGGCGCAAACAGGTATCCCT (BglⅡ) | This study |
| 1821upF | GGTTACCCGCATGCAAGATCTCTGGTAGTTAACCTGTGCATACAACAA (BglⅡ) | This study |
| 1821upR | TCGCGCGAGTCCAGAGCGGTGTTATTCAGC | This study |
| 1821downF | CTCTGGACTCGCGCGATCTGGACAAGTT | This study |
| 1821downR | GGGGCCCTTCTAGATAGATCTCGAGCAGTTCCGTTTTGTGATGCC (BglⅡ) | This study |
| 2289upF | GGTTACCCGCATGCAAGATCTGGGTGGCGTGCAGCGATTCAT (BglⅡ) | This study |
| 2289upR | GGAATCGGCGGACATCATCAAGGGAAACCG | This study |
| 2289downF | GATGTCCGCCGATTCCGATGCGTCCGGA | This study |
| 2289downR | GGGGCCCTTCTAGATAGATCTGGGGAGCGCGGCCGAATA (BglⅡ) | This study |
| 2291upF | GGTTACCCGCATGCAAGATCTGCATGAAGTTATAGCAGACGGTATCGA (BglⅡ) | This study |
| 2291upR | ACAAAGCGGCGATCGTAGGAGGGGCGCTG | This study |
| 2291downF | ACGATCGCCGCTTTGTCGAGGCGGTAAC | This study |
| 2291downR | GGGGCCCTTCTAGATAGATCTGCTGGCCGGCGAAGCAACCCA (BglⅡ) | This study |
| 2977upF | GGTTACCCGCATGCAAGATCTGACCGCGGCGATCAGGTCGA (BglⅡ) | This study |
| 2977upR | AGGTAGAAGCGATCAGAGAAAGCACGGCTG | This study |
| 2977downF | CTGATCGCTTCTACCTGGCGCTGATCGTCG | This study |
| 2977downR | GGGGCCCTTCTAGATAGATCTCCTGTCCCCTATCGGCGATG (BglⅡ) | This study |
| 3323upF | GGTTACCCGCATGCAAGATCTGCTCTGGCTTACCTGCTGGCG (BglⅡ) | This study |
| 3323upR | TCCAGGGGTGCCACCTGCATCGCGCTCA | This study |
| 3323downF | AGGTGGCACCCCTGGAAGAGGCGCTGGCA | This study |
| 3323downR | GGGGCCCTTCTAGATAGATCTCCATTCCATCTCTCCGTGGTGAAAC (BglⅡ) | This study |
| 0310comF | GGAGATATACATATGGATATCATGCTAGTTCTAGAACTGGTAATTGTTCT (EcoRⅤ) | This study |
| 0310comR | GTGGTGGTGCTCGAGGATATCCAGCATAACGCTGCCCAGC (EcoRⅤ) | This study |
| 0790comF | GGAGATATACATATGGATATCATGATCCAGGTGATTATCGCGC (EcoRⅤ) | This study |
|  |  |  |
| **TABLE S2** Continued | |  |
| 0790comR | GTGGTGGTGCTCGAGGATATCGTAGAACAGGCCGATCAGCG (EcoRⅤ) | This study |

**TABLE S3** List of the significantly changed DAPs. The protein ID, gene ID, annotation, matched proteins, matched peptides, razor unique peptides, unique peptides, coverage, molecule weight, LFQ intensity, and fold change in H_2_O_2_ group vs. control group of the significantly changed DAPs are shown.

Please see the attached Excel file TABLE S3.

**TABLE S4** List of the DAPs only detected in H_2_O_2_ group or control group. The protein ID, gene ID, annotation, matched proteins, matched peptides, razor unique peptides, unique peptides, coverage, molecule weight, and LFQ intensity of the DAPs only detected in H_2_O_2_ group or control group are shown.

Please see the attached Excel file TABLE S4.

**TABLE S5.** Top 20 enriched GO pathways of the DAPs in H_2_O_2_ group *vs*. control group. Up and down arrows indicate upregulation and downregulation, respectively. The *p*-value was set as < 0.05.

| **GO team** | **Hits** | ***p*-value** | **Proteins (H_2_O_2_ *vs*. Control)** | **Rich factor** |
| --- | --- | --- | --- | --- |
| Macromolecule transmembrane transporter activity | 4 | 0.002352 | ACY85486.1(↑), ACY84788.1(↓), ACY83017.1(↓),  ACY83483.1(↑) | 0.363636 |
| Glucuronate metabolic process | 2 | 0.003174 | ACY83283.1(↑), ACY85124.1(↑) | 1 |
| Uronic acid metabolic process | 2 | 0.003174 | ACY83283.1(↑), ACY85124.1(↑) | 1 |
| Glucuronate catabolic process | 2 | 0.003174 | ACY83283.1(↑), ACY85124.1(↑) | 1 |
| C4-dicarboxylate transport | 2 | 0.009168 | ACY83157.1(↑), ACY83635.1(↑) | 0.666667 |
| Pore complex | 2 | 0.009168 | ACY84663.1(↑), ACY84082.1(↑) | 0.666667 |
| C4-dicarboxylate transmembrane transporter activity | 2 | 0.009168 | ACY83157.1(↑), ACY83635.1(↑) | 0.666667 |
| Wide pore channel activity | 2 | 0.017658 | ACY84663.1(↑), ACY84082.1(↑) | 0.5 |
| Monosaccharide catabolic process | 2 | 0.017658 | ACY83283.1(↑), ACY85124.1(↑) | 0.5 |
| Dicarboxylic acid transport | 2 | 0.017658 | ACY83157.1(↑), ACY83635.1(↑) | 0.5 |
| Dicarboxylic acid transmembrane transporter activity | 2 | 0.017658 | ACY83157.1(↑), ACY83635.1(↑) | 0.5 |
| Porin activity | 2 | 0.017658 | ACY84663.1(↑), ACY84082.1(↑) | 0.5 |
| Cell communication | 8 | 0.027534 | ACY83519.1(↑), ACY84343.1(↑), ACY84592.1(↑),  ACY85439.1(↑), ACY85552.1(↑), ACY84565.1(↓),  ACY82991.1(↑), ACY85694.1(↑) | 0.123077 |
| Secretion by cell | 2 | 0.028349 | ACY83017.1(↓), ACY85486.1(↑) | 0.4 |
| Secretion | 2 | 0.028349 | ACY83017.1(↓), ACY85486.1(↑) | 0.4 |
| Protein secretion | 2 | 0.028349 | ACY83017.1(↓), ACY85486.1(↑) | 0.4 |
| Peptide secretion | 2 | 0.028349 | ACY83017.1(↓), ACY85486.1(↑) | 0.4 |
|  |  |  |  |  |
| **TABLE S5** Continued |  |  |  |  |
| Integral component of membrane | 25 | 0.037717 | ACY83483.1(↑), ACY85486.1(↑), ACY84663.1(↑),  ACY84082.1(↑), ACY83352.1(↑), ACY82991.1(↑),  ACY83017.1(↓), ACY86145.1(↓), ACY83121.1(↑),  ACY83157.1(↑), ACY83635.1(↑), ACY83886.1(↑),  ACY84285.1(↑), ACY84368.1(↑), ACY84534.1(↓),  ACY84565.1(↓), ACY84592.1(↑), ACY84702.1(↑),  ACY84997.1(↑), ACY85267.1(↓), ACY85466.1(↓),  ACY85552.1(↑), ACY85686.1(↓), ACY86227.1(↑),  ACY83108.1(↑) | 0.080128 |
| Intrinsic component of membrane | 25 | 0.040413 | ACY82991.1(↑), ACY83017.1(↓), ACY86145.1(↓),  ACY83121.1(↑), ACY83157.1(↑), ACY83635.1(↑),  ACY83886.1(↑), ACY84285.1(↑), ACY84368.1(↑),  ACY84534.1(↓), ACY84565.1(↓), ACY84592.1(↑),  ACY84702.1(↑), ACY84997.1(↑), ACY85267.1(↓),  ACY85466.1(↓), ACY85552.1(↑), ACY85686.1(↓),  ACY86227.1(↑), ACY83108.1(↑), ACY83483.1(↑),  ACY85486.1(↑), ACY84663.1(↑), ACY84082.1(↑),  ACY83352.1(↑) | 0.079618 |
| Oxidoreductase activity, acting on other nitrogenous compounds as donors | 2 | 0.040968 | ACY83098.1(↓), ACY85119.1(↓) | 0.333333 |

**TABLE S6.** Top 10 enriched KEGG pathways of the DAPs in H_2_O_2_ group *vs.* control group. Up and down arrows indicate upregulation and downregulation, respectively.

| **KEGG Pathway** | **Hits** | ***p*-value** | **Proteins (H_2_O_2_ *vs*. Control)** | **Rich factor** |
| --- | --- | --- | --- | --- |
| Pentose and glucuronate interconversions | 3 | 0.015795 | ACY85126.1(↑), ACY83283.1(↑), ACY85124.1(↑) | 0.3 |
| Arginine biosynthesis | 3 | 0.020836 | ACY86007.1(↓), ACY86311.1(↓), ACY86078.1(↑) | 0.272727 |
| Pantothenate and CoA biosynthesis | 3 | 0.040619 | ACY83497.1(↑), ACY82941.1(↓), ACY83473.1(↓) | 0.214286 |
| Tryptophan metabolism | 2 | 0.07102 | ACY86309.1(↑), ACY84767.1(↓) | 0.25 |
| Valine, leucine and isoleucine biosynthesis | 2 | 0.088018 | ACY82941.1(↓), ACY83473.1(↓) | 0.222222 |
| Biosynthesis of vancomycin group antibiotics | 1 | 0.109976 | ACY82952.1(↑) | 0.5 |
| One carbon pool by folate | 2 | 0.144688 | ACY84590.1(↑), ACY84675.1(↓) | 0.166667 |
| Polyketide sugar unit biosynthesis | 1 | 0.160381 | ACY82952.1(↑) | 0.333333 |
| Acarbose and validamycin biosynthesis | 1 | 0.160381 | ACY82952.1(↑) | 0.333333 |
| Two-component system | 8 | 0.192975 | ACY83519.1(↑), ACY84592.1(↑), ACY83098.1(↓),  ACY85439.1(↑), ACY85552.1(↑), ACY84082.1(↑),  ACY86227.1(↑), ACY84788.1(↓), | 0.080808 |
